# Supplementary material for: Global changes of the RNA-bound proteome during the maternal-to-zygotic transition in Drosophila
Source: Nat Commun. 2016 Jul 5;7:12128. doi: 10.1038/ncomms12128 (PMC4935972; doi:10.1038/ncomms12128)
Supplement: Supplementary Information — Supplementary Figures 1-4, Supplementary Tables 1-2, Supplementary Notes 1-3 and Supplementary References [file ncomms12128-s1.pdf]

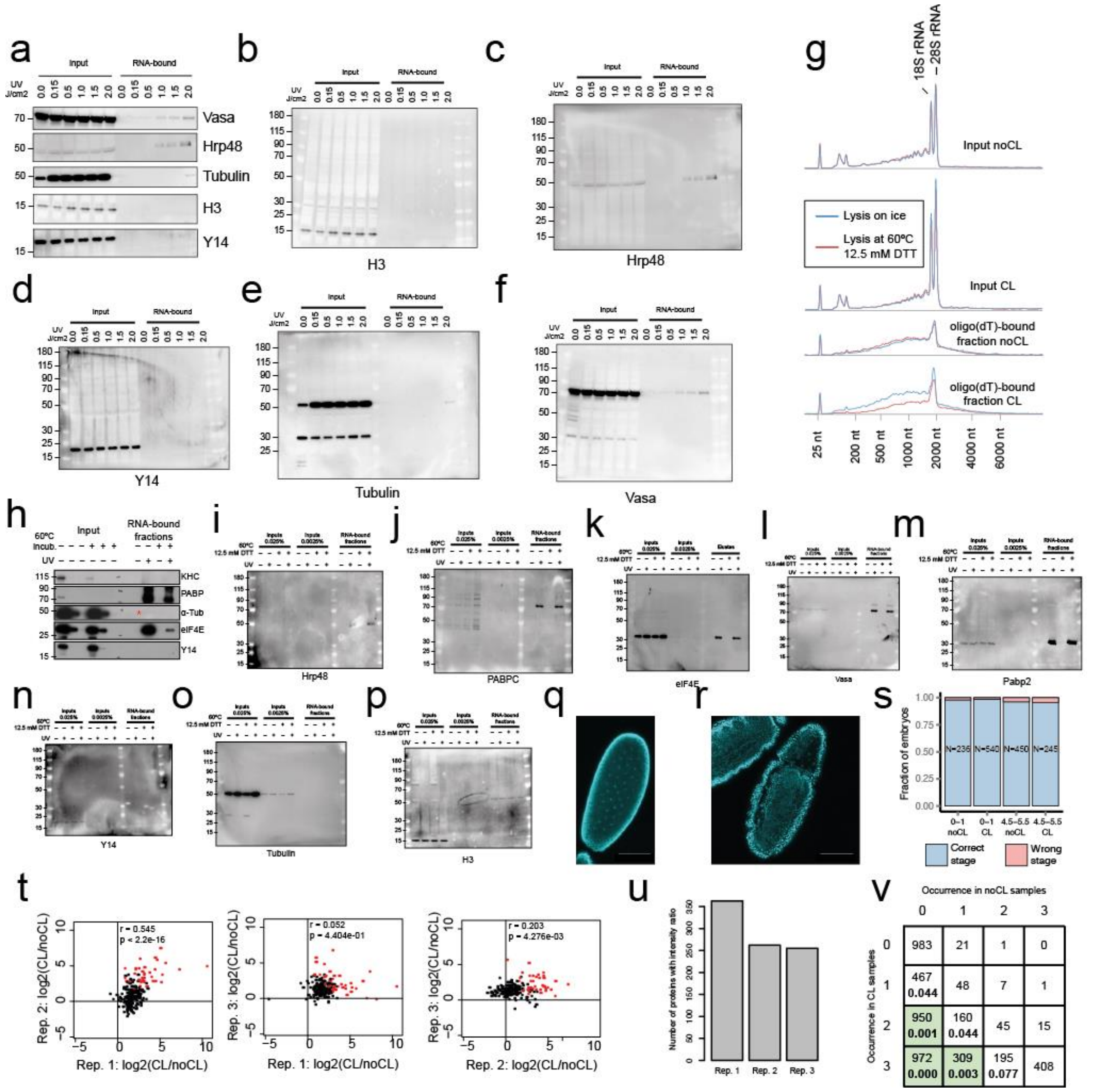

Supplementary Fig. 1 Additional data on identification of the *Drosophila* interactome, related to Figure 1

(a) Western blot analysis of oligo(dT) bound fractions recovered from embryos that received different UV doses (0–2.0 J/cm^2). Yields of known RBPs Vasa and Hrp48 are UV-dependent. Histone H3, tubulin and Y14 were used as a measure of contamination. 1.0 J/cm^2 was used in further experiments. **(b-f)** Full-size blots, related to Supplementary Fig. 1a. **(g)** RNA profiles of total embryo lysates and oligo(dT) bound fractions of embryos lysed at room temperature (blue) and 60ºC/12.5 mM DTT (red) determined by

Bioanalyzer 2100. **(h)** An example of western-blot analysis of interactome samples captured from embryos lysed at room temperature and 60ºC/12.5 mM DTT. **(i-p)** Full-size blots, related to Fig. 1e. **(q,r)** Images of DAPI stained 0–1 h (q) and 4.5–5.5 h (r) embryos. Scale bars indicate 100 μm. **(s)** Numbers of embryos at correct and wrong stages in the samples. **(t)** Scatter plots comparing protein abundance ratios CL/noCL in three biological replicates. Red dots – significantly enriched proteins. **(u)** Numbers of proteins with non-infinite intensity ratios. **(v)** Numbers of peptides with different occurrences in CL and noCL samples and estimated FDRs. Green cells represent peptides with less than 0.01 false positives.

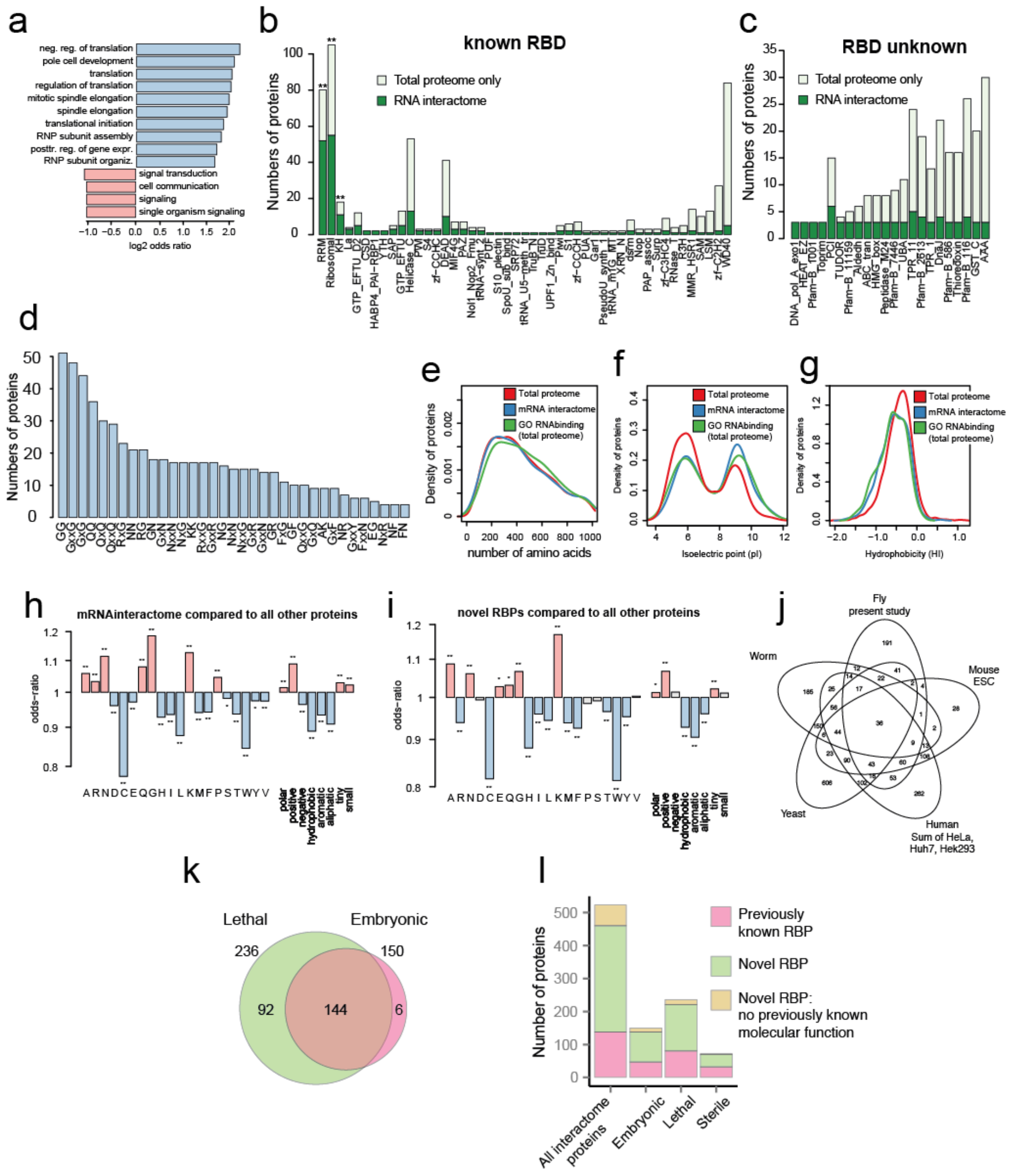

Supplementary Fig. 2 Data supporting the analysis of the *Drosophila* interactome, related to Figure 2

(a) GO-terms related to biological process enriched (blue) and depleted (red) in the interactome as compared to the total proteome. **(b)** Numbers of proteins harboring one of the 47 Pfam-annotated RBDs identified in the interactome and the proteome. RRM, ribosomal and KH domains are statistically enriched

in the interactome. **(c)** Numbers of proteins containing one of the non-RBD domains identified in the interactome. **(d)** Numbers of interactome proteins containing various repetitive sequence patterns. **(e)** Density of calculated lengths of proteins identified in the total proteome (red), mRNA interactome (blue) and proteins identified in the total proteome and GO annotated as RNA binding. **(f)** Density of calculated isoelectric points (pI) in the three protein groups defined in (e). **(g)** Density of calculated hydrophobicity values in the three protein groups defined in (e). **(h)** Enrichment of amino acids and amino acid classes in the mRNA interactome, compared the proteome. **(i)** Enrichment of amino acids and amino acid classes among the newly discovered RBPs, compared to the proteome. **(j)** Venn diagram comparing RNA interactomes of five organisms: the fruit fly (this study), the worm, mouse and human (total of HEK293, Huh7 and HeLa cells). **(k)** Venn diagram comparing numbers of interactome proteins belonging to “lethal” and “embryonic” phenotypic classes. **(l)** Numbers of previously known RBPs (pink), novel RBPs with a previously known molecular function (green) and novel RBPs for which no molecular function was previously known (yellow).

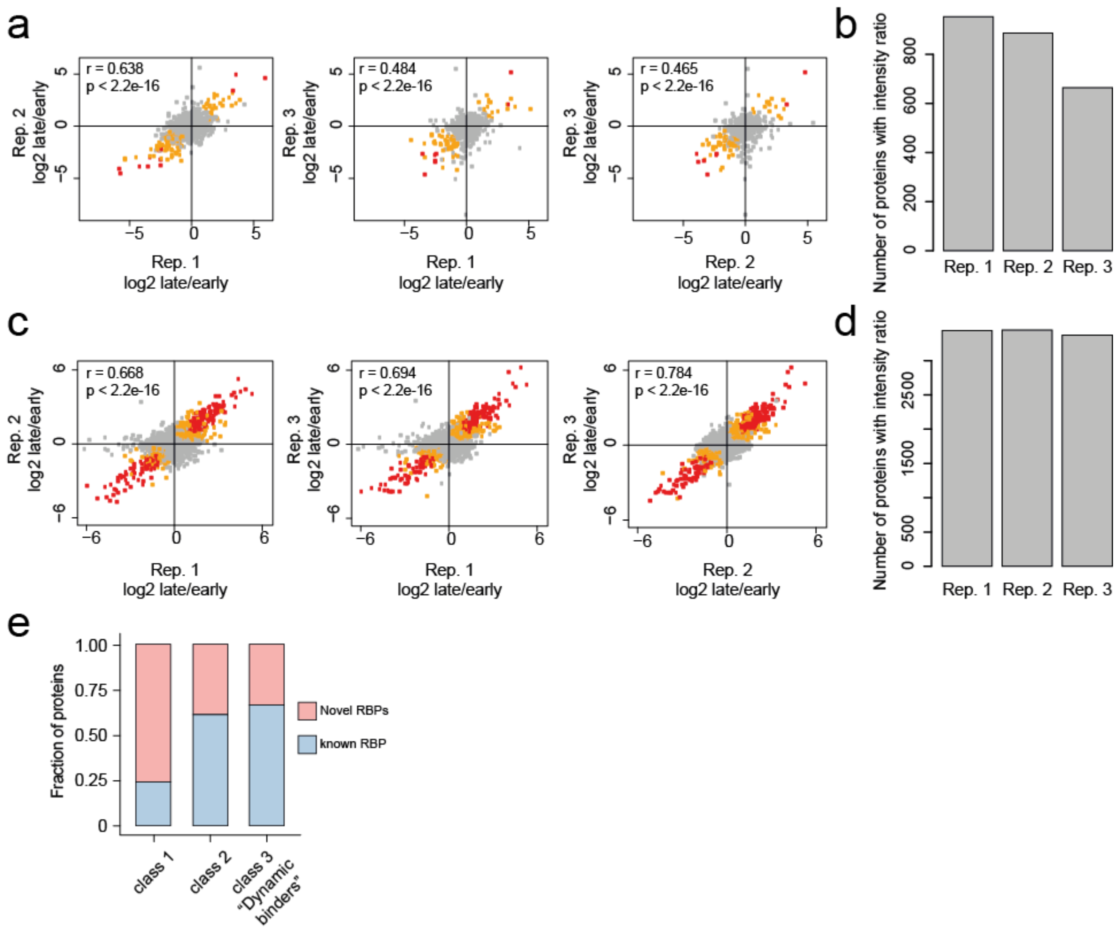

Supplementary Fig. 3 Additional data related to the study of interactome dynamics during the MZT, related to Figure 4

(a) Ratios of protein abundance late/early in RNA bound fractions. **(b)** Numbers of proteins with intensity ratio in RNA bound fractions, in each of the three biological replicates. **(c)** Ratios of protein abundance late/early in total embryo lysates. **(d)** Numbers of proteins with intensity ratio in total embryo lysates, in each of the three biological replicates. **(e)** Distribution of previously known (blue) and novel (pink) RBPs in the three dynamic RBP classes determined in Fig. 4f. Only RNA interactome proteins were considered.

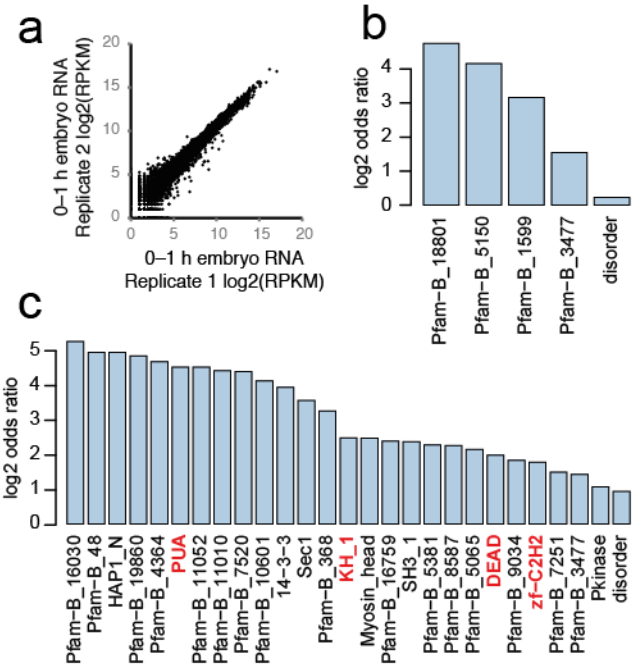

Supplementary Fig. 4 Additional data related to possible explanations of the dynamic activity of some RBPs

(a) Scatter plot showing gene expression levels (RPKM) in 0–1 h mbryos in two biological replicates. **(b)** Pfam domains enriched in alternatively used exons that are upregulated 4.5–5.5 h embryos (and, respectively, downregulated in 0–1 h embryos). **(c)** Pfam domains enriched in alternatively used exons that are downregulated in 4.5–5.5 h embryos (and, respectively, upregulated in 0–1 h embryos).

Supplementary Table 1. Oligonucleotides used for qPCR quantification of RNAs.

Target	Forward primer	Reverse primer
18S rRNA [1]	CGGAGAGGGAGCCTGAGAA	AGCTGGGAGTGGGTAATTTACG
ts mRNA	ACACGTCTACCTGAACCACG	GATGTCCTGCACCTGACGTT
gapdh mRNA	TTCACCACCATTGACAAGGC	CTTCATGTCGGGGCTGTAGG

Supplementary Table 2. Antibodies used for immunoblotting.

Antigen	Source animal	Dilution	Source
α-Tubulin	Mouse	1:20000	Sigma Aldrich
Kinesin heavy chain	Rabbit	1:3000	Cytoskeleton Inc.
PABP	Rabbit	1:5000	Gift of M. Hentze [2]
Hrp48	Rabbit	1:1000	Gift of D. Rio [3]
eIF4E	Rabbit	1:3000	Gift of A. Nakamura [4]
Y14	Rat	1:2500	A.E. [5]
H3	Rabbit	1:2500	Abcam
Vasa	Rat	1:2000	A.E. [6]
GFP	Rabbit	1:5000	Abcam

Supplementary Note 1. Choice of the UV dose for cross-linking RBPs in *Drosophila* embryos

We selected 1.0 J/cm2 as the minimum UV dose required to promote efficient protein RNA crosslinking without causing RNA degradation in embryos. Although irradiation with 2.0 J/cm2 in some instances resulted in slightly higher crosslinking efficiency, occasionally it also resulted in tubulin contamination of the UV cross-linked samples (see new Supplemental Fig. 1a). Additionally, in order to achieve a dose of 2.0 J/cm2, the duration of UV exposure had to be doubled (compared with 1.0 J/cm2). Assuming that shorter embryo processing times would favor a higher quality of the lysates, and given that UV irradiation with 1.0 J/cm2 was sufficient to precipitate enough protein for MS analysis, we opted for the lower dose. Finally, the shorter time enabled us to generate multiple samples (all steps, from embryo collection to obtention of lysates) in one embryo collection day. This is crucial, given that the Drosophila embryo collection cages are populated with adult flies and produce embryos only a few days per month. For these reasons we considered the UV dose of 1.0 J/cm2 to be a good compromise between irradiation time and crosslinking efficiency.

Supplementary note 2: Expanded description of mass spectrometry and data analysis

Sample preparation for MS

Samples were processed according to the standard filter aided sample preparation protocol [7] with minor modifications. Cysteines were reduced (10 mM DTT, 55˚C, 30 min) and alkylated (20 mM Iodoacetamide, 30 min in the

dark). Samples were buffer-exchanged into a buffer containing 8 M Urea and 50 mM TEAB (Triethylammoniumbicarbonate, Sigma-Aldrich) using 10 kDa centrifugal filters (Millipore) and incubated with 1 µg sequencing grade Lys-C (Promega) at 37˚C for 4 h. The buffer was diluted with 50 mM TEAB to a final Urea concentration of 2 M and incubated for another 12 h at 37˚C. Resulting peptides were desalted using OASIS HLB solid phase extraction cartridges (Waters) and dried in a vacuum concentrator. Peptides were labelled using isobaric 6plex tandem mass tags (TMT, Life technologies) [8] as previously described [9]. Labelling efficiency was assessed by LC-MS/MS and samples from total proteome, interactome and comparative interactome, respectively, were mixed in approximately equal amounts based on median peptide intensity. The resulting complex peptide mixtures were fractionated by offline high pH reversed phase chromatography [10]. Briefly, peptides were resuspended in buffer A (20 mM Ammoniumformate, pH 10) and fractionated over a Gemini 3U C18 110A column (100 x 1.00 mm, Phenomenex) using a 60 min linear gradient from 0-35% solvent B (100% acetonitrile) at a constant flow rate of 0.1 mL/min. Resulting fractions were combined into 8-9 subfractions, desalted, dried in a vacuum concentrator and reconstituted in 5% DMSO 1% formic acid.

LC-MS/MS

Samples were analyzed on a LTQ-Orbitrap Velos Pro mass spectrometer (Thermo Scientific) coupled to a nanoAcquity UPLC system (Waters). Peptides were loaded onto a trapping column (nanoAcquity Symmetry C_{18}, 5 µm, 180 µm × 20 mm) at a flow rate of 15 µl/min with solvent A (0.1% formic acid). Peptides were separated over an analytical column

(nanoAcquity BEH C18, 1.7 µm, 75 µm × 200 mm) at a constant flow of 0.3 µl/min using the following gradient: 3% solvent B (acetonitrile, 0.1% formic acid) for 10 min, 7-25% solvent B within 160 min, 25-40% solvent B within 10 min, 85% solvent B for 6 min. Peptides were introduced into the mass spectrometer using a Pico-Tip Emitter (360 µm outer diameter × 20 µm inner diameter, 10 µm tip, New Objective). For peptide identification and quantification a MS3 method was set up [11]. MS survey scans were acquired from 350–1500 *m*/*z* at a nominal resolution of 30,000. Lock mass correction was enabled using the singly charged polysiloxane ion (*m*/*z* 445.12003). The 15 most abundant peptides were isolated within a 2 Da window and subjected to MS2 sequencing using collision-induced dissociation in the ion trap (activation time 20 ms, normalized collision energy 35%). Singly charged and unassigned charge states were excluded from analysis. Precursors were dynamically excluded for 45 s (exclusion list size was set to 500). The most abundant product ion between 400-800 *m*/*z* from each MS2 scan was subjected to MS3 analysis. The isolation window was set to 4 Da. HCD normalized collision energy was set to 55%. Fragment ions were recorded in the Orbitrap at a resolution of 15,000.

Peptide identification and quantification

Raw data were processed with Proteome Discoverer 1.4 (Thermo Scientific). MS/MS spectra were filtered to retain the 10 most abundant ions for each 100 Da window and searched against the Drosophila UniProt database (version 2014_06) concatenated to a database containing protein sequences of common contaminants using Mascot 2.2 (Matrix Science). Enzyme cleavage specificity was set to LysC, allowing a maximum of two

missed cleavages. Cysteine carbamidomethylation was set as fixed modification, and oxidation (M) and TMT6x (N-term, K) were used as variable modifications. The peptide mass tolerances were set to 10 ppm for MS, and 0.5 Da for MS/MS. Percolator was used for false discovery rate determination. Only peptides passing the following filters were retained: 1% false discovery rate, minimum peptide length = 6, minimum peptide ion score = 20, search engine rank = 1. Reporter ion quantification was performed by the reporter ion quantifier node within Proteome Discoverer using a peak integration tolerance of 20 ppm. Raw reporter ion intensities for each peptide were used for further bioinformatics analyses.

Peptides were mapped back on the Uniprot protein sequences. For each Flybase gene identifier a generic protein was selected. Among all proteins that cover most peptides, the longest was selected. Only peptides uniquely mapping to one protein model were considered for the analysis. A quantitative differential proteome analysis was performed for the mRNA interactome analysis (comparing CL and noCL) for the differential total proteome analysis (comparing 4-5h and 0-1h) and the differential binding analysis (comparing 4-5h and 0-1h). In the case of multiple quantification events per peptide, the event with largest total intensity over all samples was considered. The ratios of peptide intensities between CL and noCL samples were computed. For each protein, intensity log-ratios were averaged over all peptides. Protein intensity log-ratios were tested against the null hypothesis that log-ratios are equal to zero using a moderated t-test [12] implemented in the R/Bioconductor package limma [13]. p-values were corrected for multiple

testing by controlling the false discovery rate (FDR) using the method of Benjamini-Hochberg [14].

In the case of the RNA interactome, quantitative analysis could only be performed for a low number of proteins because of lack of values in the noCL control, due the low background. Therefore, a second, semi-quantitative approach was applied assuming that peptides without quantitative information are below the detection limit. The number of replicates in which a peptide had been identified was used as a semi-quantitative measure. In total this allows classification of peptides into 16 different groups, as represented in Supplementary Fig. 1v

The FDRs were estimated as ratios resulting from division of the transposed matrix in Supplementary Fig. 1v by itself. The following example illustrates this approach. There are 160 peptides that occur in two CL replicates and one noCL replicate, and 7 peptides that occur in one CL and two noCL replicates. FDR for the aforementioned 160 peptides is estimated as 7/160= 0.04375. Since only peptides for which FDR<0.01 were considered high confidence hits (green cells Supplementary Fig. 1v), the aforementioned 160 peptides were not considered high confidence hits. Only proteins comprising peptides with FDR<0.01 were included in the *Drosophila* RNA interactome.

To identify dynamic RNA-binding proteins, the quantitative analysis of the differential binding was compared to the differential total proteome. The differentially binding proteins at FDR 10% were separated in two classes,

those that change binding, because of a change in protein abundance, and those that change in binding but do not change protein abundance. The latter we called “dynamic binders”. To identify a set of proteins not changing in abundance, we selected all proteins above an FDR threshold of 43.6%. For a set of proteins one can estimate the absolute number of changing proteins by subtracting the expected false discoveries from the size of the set. The threshold is chosen such that the absolute number of changing proteins is maximized.

GO and Pfam annotation for proteins was obtained from Ensembl 75. Proteins were classified as known RNA-binding proteins using the GO-term “RNA-binding” and RNA-related by a manually selected list of GO-terms related to RNA biology. A manually curated list of Pfam RNA-binding domains was used to classify proteins as harboring a known RBD. Gene set enrichment analysis for GO categories and Pfam domains was performed using Fisher’s exact test. P-values were corrected for multiple testing by the method of Benjamini-Hochberg [14].

Phenotype information was obtained from FlyBase release 2015_02.

Supplementary note 3. Enrichment of disordered, repetitive and low complexity regions in interactome proteins as compared to the total embryo lysate, related to Fig. 2c.

All interactome proteins (blue lines on Fig. 2c), previously known (green) and novel (purple) RBPs were tested for enrichment of disordered,

repetitive or low complexity regions over the total embryo lysate using the two-sample Kolmogorov-Smirnov test. Results are presented in the table:

Sample	p-value
**Disordered regions**	
Whole interactome	8.196e-08
Previously known RBPs	3.031e-11
Novel RBPs	0.3829
**Repetitive regions**	
Whole interactome	3.445e-06
Previously known RBPs	1.391e-10
Novel RBPs	0.9783
**Low complexity regions**	
Whole interactome	1.226e-05
Previously known RBPs	4.962e-13
Novel RBPs	0.8247

Supplementary references

1. Duncan, K. E., Strein, C. & Hentze, M. W. The SXL-UNR corepressor complex uses a PABP-mediated mechanism to inhibit ribosome recruitment to msl-2 mRNA. *Molecular Cell* **36,** 571–582 (2009).
2. Moretti, F., Kaiser, C., Zdanowicz-Specht, A. & Hentze, M. W. PABP and the poly(A) tail augment microRNA repression by facilitated miRISC binding. *Nat. Struct. Mol. Biol.* **19,** 603–608 (2012).
3. Hammond, L. E., Rudner, D. Z., Kanaar, R. & Rio, D. C. Mutations in the hrp48 gene, which encodes a Drosophila heterogeneous nuclear ribonucleoprotein particle protein, cause lethality and developmental defects and affect P-element third-intron splicing in vivo. *Mol. Cell. Biol.* **17,** 7260–7267 (1997).
4. Nakamura, A., Sato, K. & Hanyu-Nakamura, K. Drosophila cup is an eIF4E binding protein that associates with Bruno and regulates oskar mRNA translation in oogenesis. *Dev. Cell* **6,** 69–78 (2004).
5. Hachet, O. & Ephrussi, A. Drosophila Y14 shuttles to the posterior of the oocyte and is required for oskar mRNA transport. *Curr. Biol.* **11,** 1666–1674 (2001).
6. Tomancak, P., Guichet, A., Zavorszky, P. & Ephrussi, A. Oocyte polarity depends on regulation of gurken by Vasa. *Development* **125,** 1723–1732 (1998).
7. Wiśniewski, J. R., Zougman, A., Nagaraj, N. & Mann, M. Universal

sample preparation method for proteome analysis. *Nat. Methods* **6,** 359–362 (2009).
8. Thompson, A. *et al.* Tandem mass tags: a novel quantification strategy for comparative analysis of complex protein mixtures by MS/MS. *Anal. Chem.* **75,** 1895–1904 (2003).
9. Altelaar, A. F. M. *et al.* Benchmarking stable isotope labeling based quantitative proteomics. *J Proteomics* **88,** 14–26 (2013).
10. Yang, F., Shen, Y., Camp, D. G. & Smith, R. D. High-pH reversed-phase chromatography with fraction concatenation for 2D proteomic analysis. *Expert Rev Proteomics* **9,** 129–134 (2012).
11. Ting, L., Rad, R., Gygi, S. P. & Haas, W. MS3 eliminates ratio distortion in isobaric multiplexed quantitative proteomics. *Nat. Methods* **8,** 937–940 (2011).
12. Lönnstedt, I. & Speed, T. Replicated Microarray Data. *Statistica Sinica* **12,** 31–46 (2002).
13. Smyth, G. K. in *Bioinformatics and Computational Biology Solutions Using R and Bioconductor* (eds. Gentleman, R., Carey, V. J., Huber, W., Irizarry, R. A. & Dudoit, S.) 397–420 (Springer-Verlag, 2005). doi:10.1007/0-387-29362-0_23
14. Benjamini, Y. & Hochberg, Y. Controlling the False Discovery Rate: A Practical and Powerful Approach to Multiple Testing. *Journal of the Royal Statistical Society* **57,** 289–300 (1995).
